# Supplementary material for: Humans and other commonly used model organisms are resistant to cycloheximide-mediated biases in ribosome profiling experiments
Source: Nat Commun. 2021 Aug 24;12:5094. doi: 10.1038/s41467-021-25411-y (PMC8384890; doi:10.1038/s41467-021-25411-y)
Supplement: Supplementary file 1 — Supplementary Information [file 41467_2021_25411_MOESM1_ESM.pdf]

Humans and other commonly used model organisms are resistant to cycloheximide-mediated biases in ribosome profiling experiments

Puneet Sharma[1,2,3,5], Jie Wu[1,2,3,4,5], Benedikt S. Nilges[1,2,4], Sebastian A. Leidel[1,2,3*]

[1]Max Planck Research Group for RNA Biology, Max Planck Institute for Molecular Biomedicine, 48149 Muenster, Germany

[2]Cells-in-Motion Cluster of Excellence, University of Muenster, 48149 Muenster, Germany

[3]Research group for RNA Biochemistry, Department of Chemistry, Biochemistry and Pharmaceutical Sciences, University of Bern, 3012 Bern, Switzerland

[4]Graduate School for Cellular and Biomedical Sciences, University of Bern, 3012 Bern, Switzerland

[5]These authors contributed equally: Puneet Sharma, Jie Wu, Benedikt S. Nilges

*Corresponding author: sebastian.leidel@unibe.ch

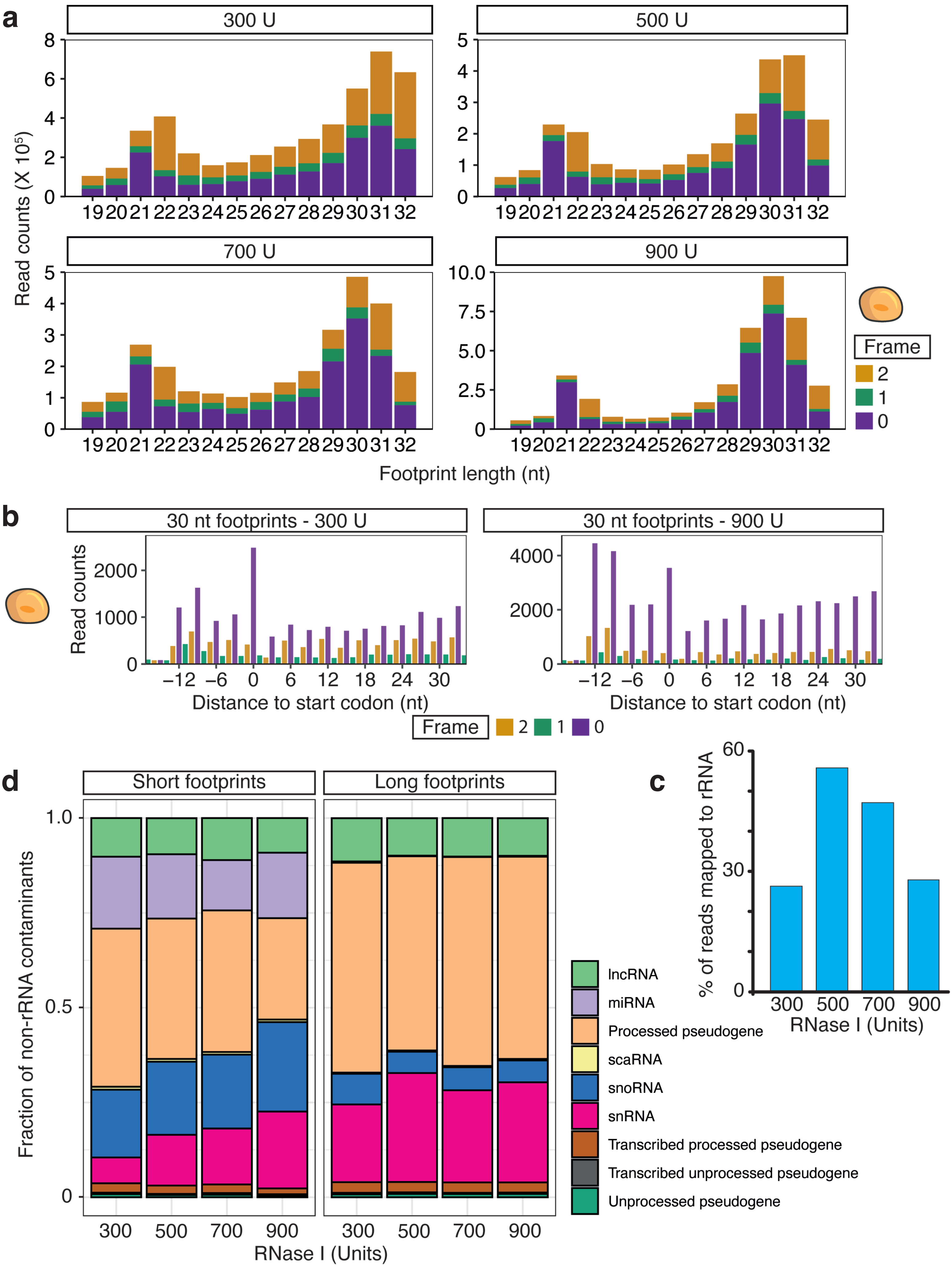

Supplementary Figure 1

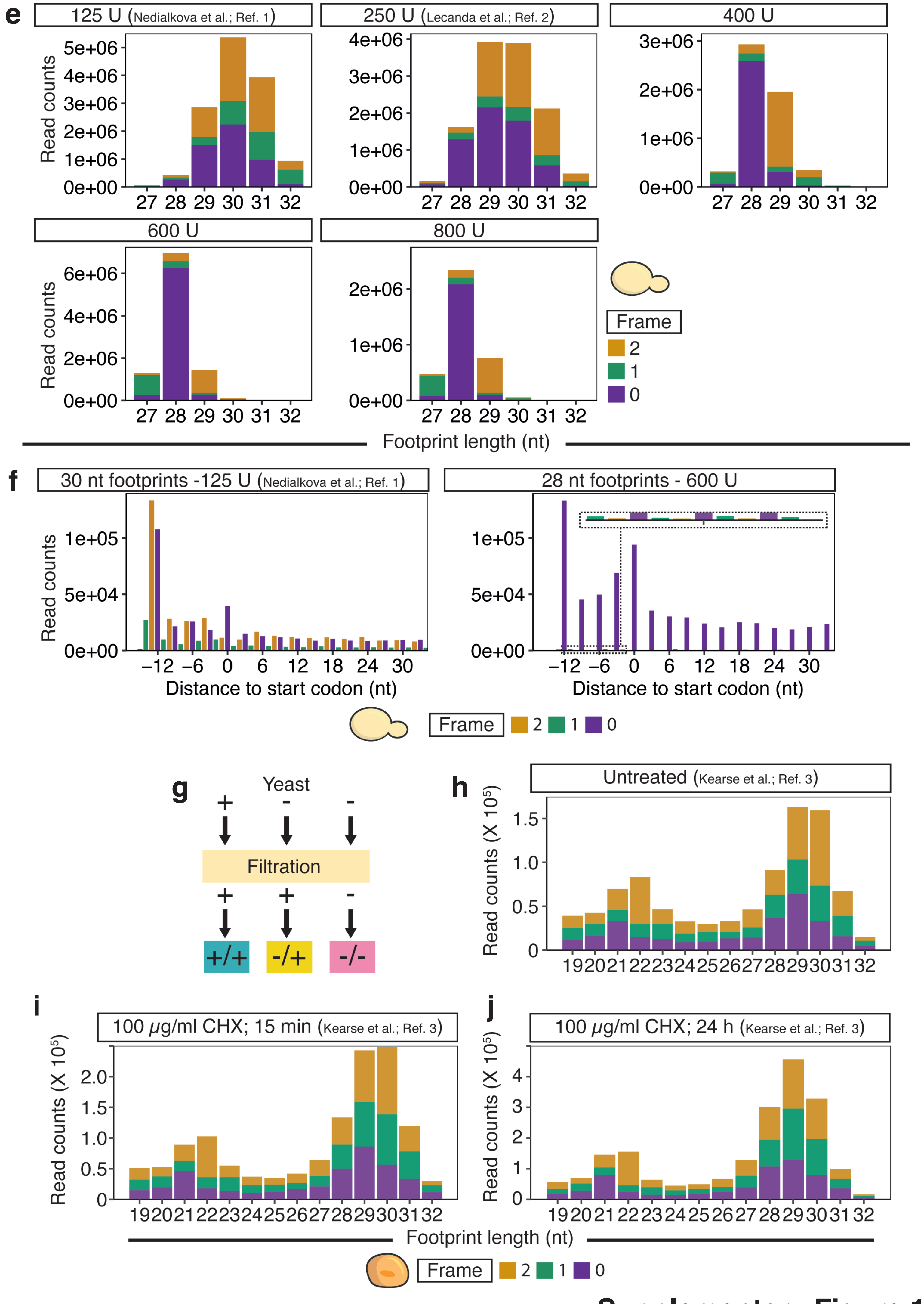

Supplementary Figure 1

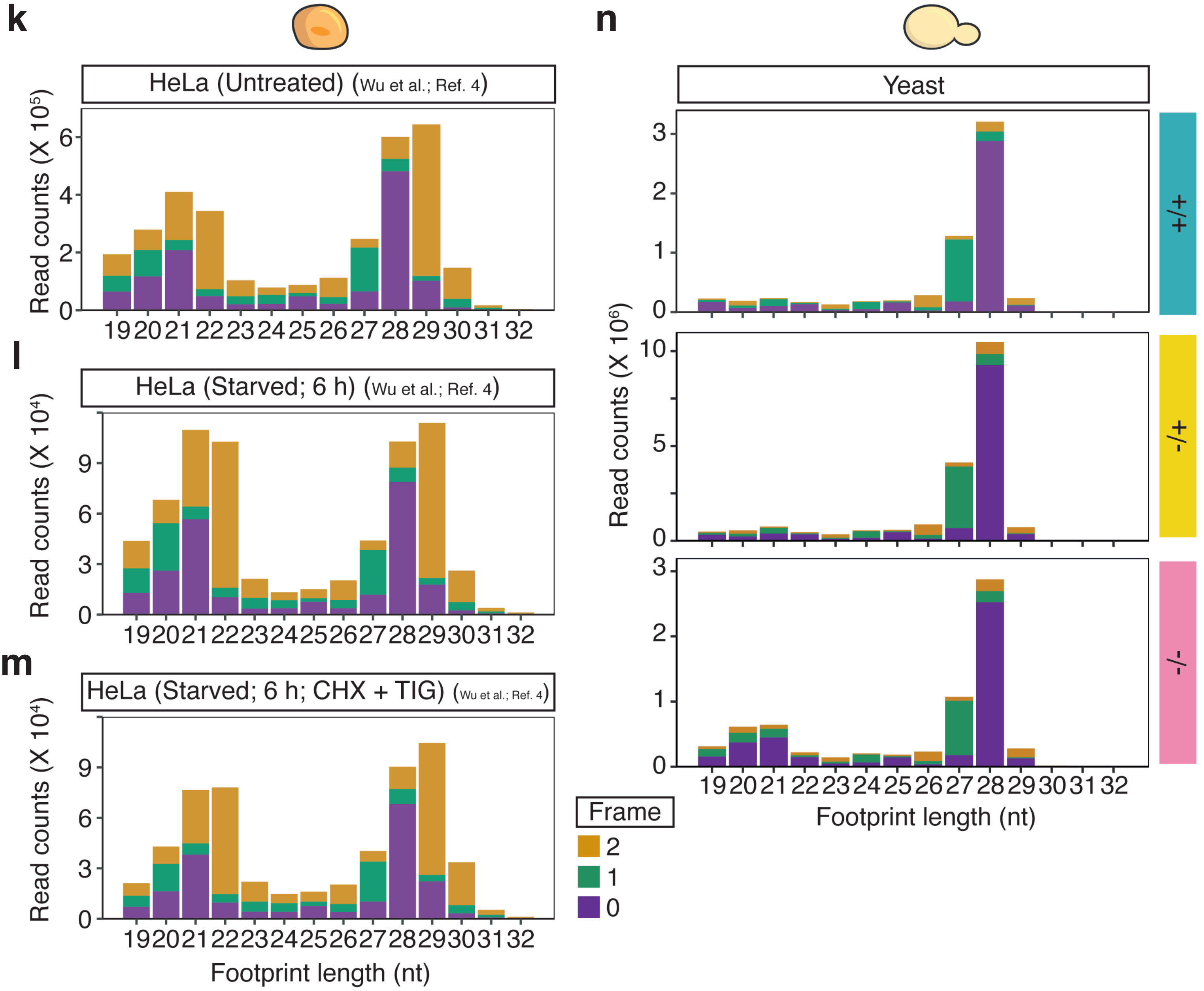

Supplementary Figure 1

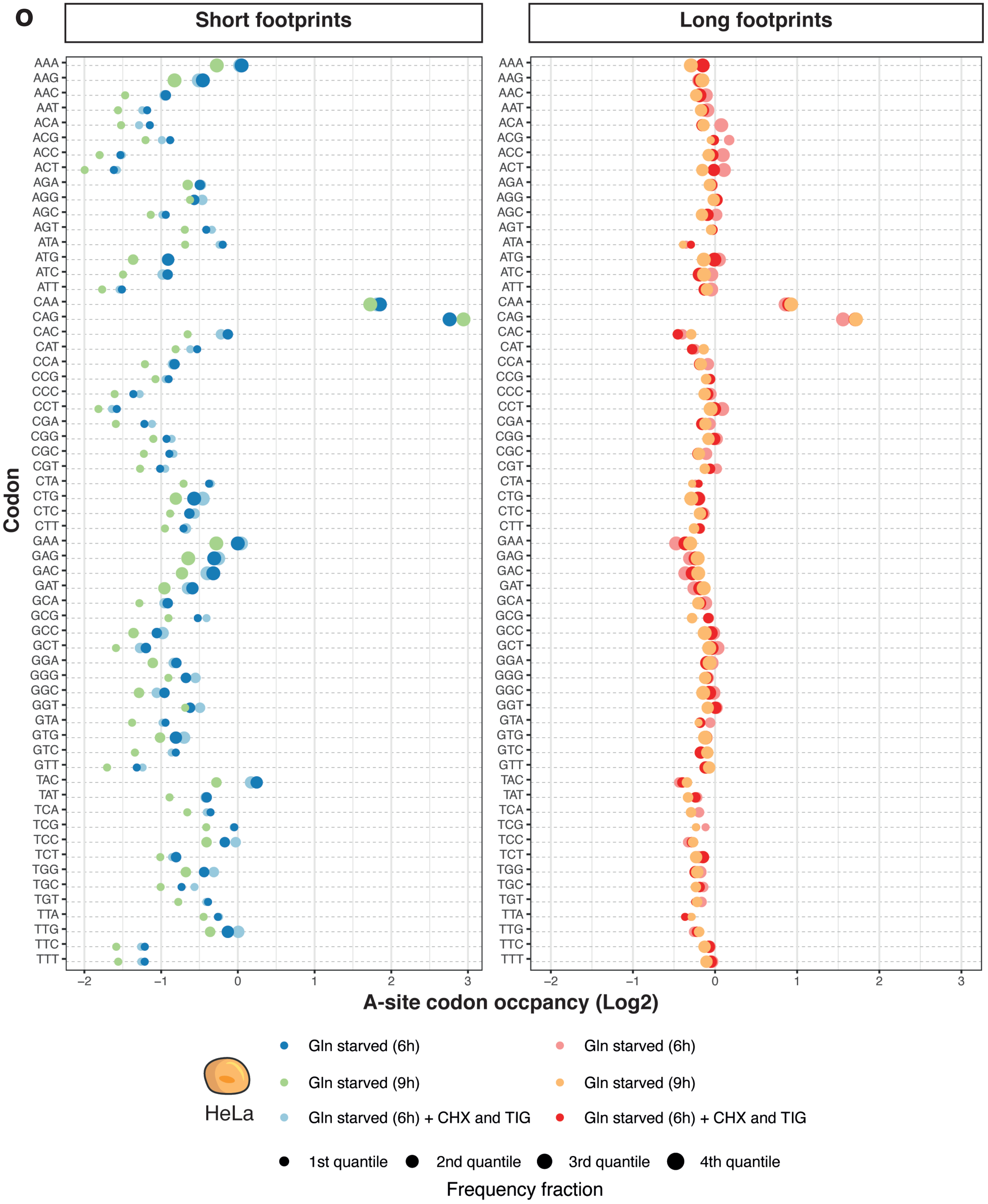

Supplementary Figure 1

Supplementary Fig. 1: Nuclease concentration used during RNase I digestion affects the footprint length distribution and reading frame. a) Histograms showing the effect of RNase I concentration on footprint length and reading frame in human -/- ribosome profiling libraries (18-32 nt footprints). b) Total number and frame of the 5' ends of ribosome footprints in a weakly (300 U) and a strongly (900 U) digested HEK 293T sample mapped to all human coding sequences. 0 represents the first nucleotide of the start codon. c) Percentage of reads mapped to rRNA in HEK 293T libraries prepared using different RNase I concentrations. d) Stacked barplots representing various non-rRNA contaminants in reads which did not map to rRNA and CDS under different RNase I concentrations. e) Same as a) for yeast +/+ libraries. Published[1,2] yeast libraries and libraries using higher RNase I concentration were generated from long (28-30 nt) footprints using a similar protocol. Plots show representative samples. f) Same as b) for yeast samples. The inset shows a magnification to visualize the low number of out-of-frame reads. g) Schematic overview of harvesting and CHX-treatment conditions used in this study in yeast. h-j) Same as a) for untreated HeLa cells (h)[3], cells treated with 100 μg/ml CHX for 15 min (i)[3] or 24 h (j)[3]. k-m) Same as a) for untreated HeLa cells (k)[4], cells starved for glutamine for 6h (l)[4] or glutamine starved cells lysed with lysis buffer containing cocktail of 100 μg/ml CHX and 100 μg/ml tigecycline (TIG) (m)[4]. n) Representative histograms showing the influence of CHX on footprint length and the reading frame in yeast libraries. Footprints were excised between 18-30 nt (yeast) and 18-32 nt (HEK 293T). o) Codon specific changes in A-site ribosome occupancy for short and long footprints in glutamine starved HeLa cells lysed with buffer containing either 100 μg/ml CHX or cocktail of 100 μg/ml CHX and 100 μg/ml TIG[4].

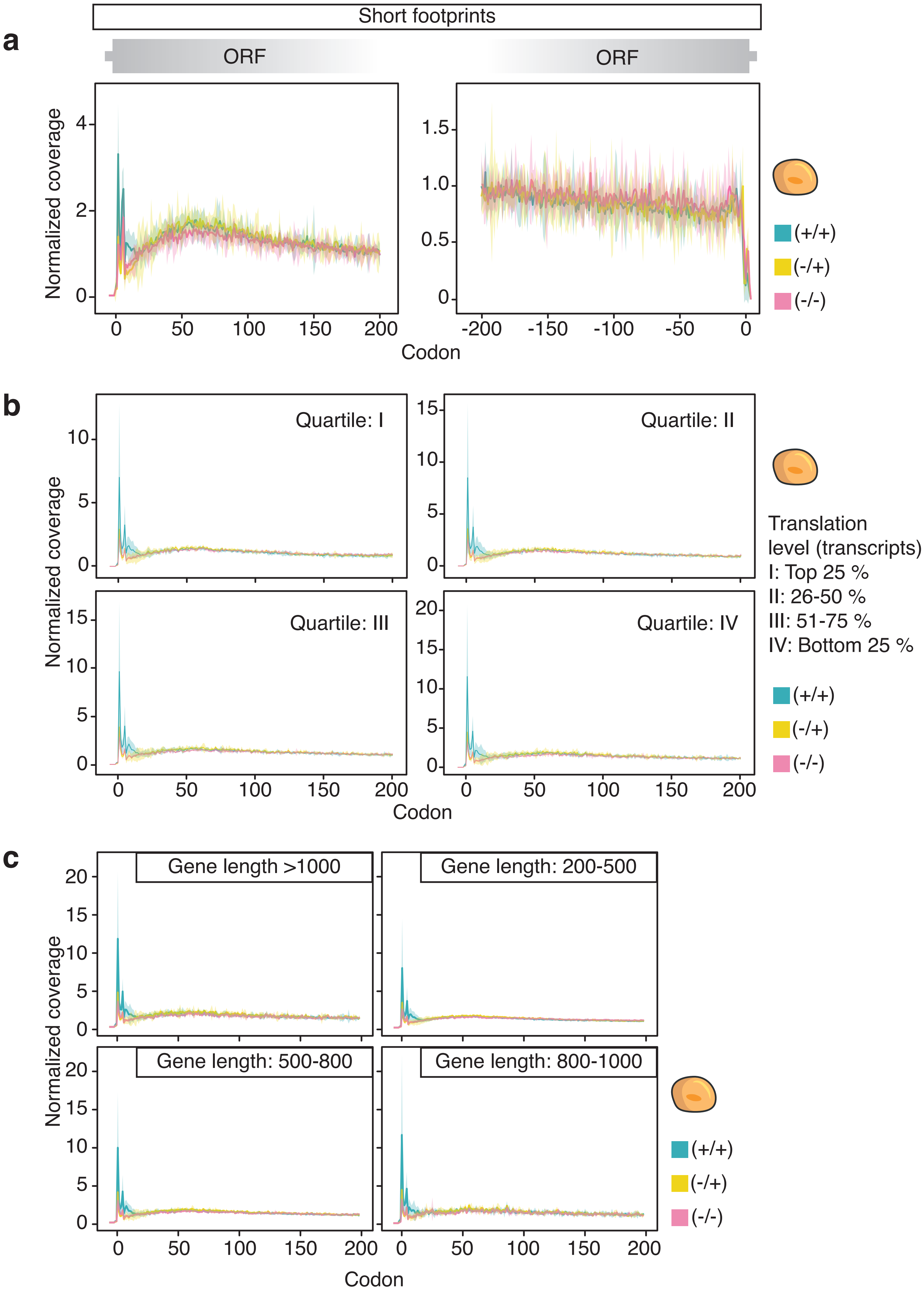

Supplementary Figure 2

d

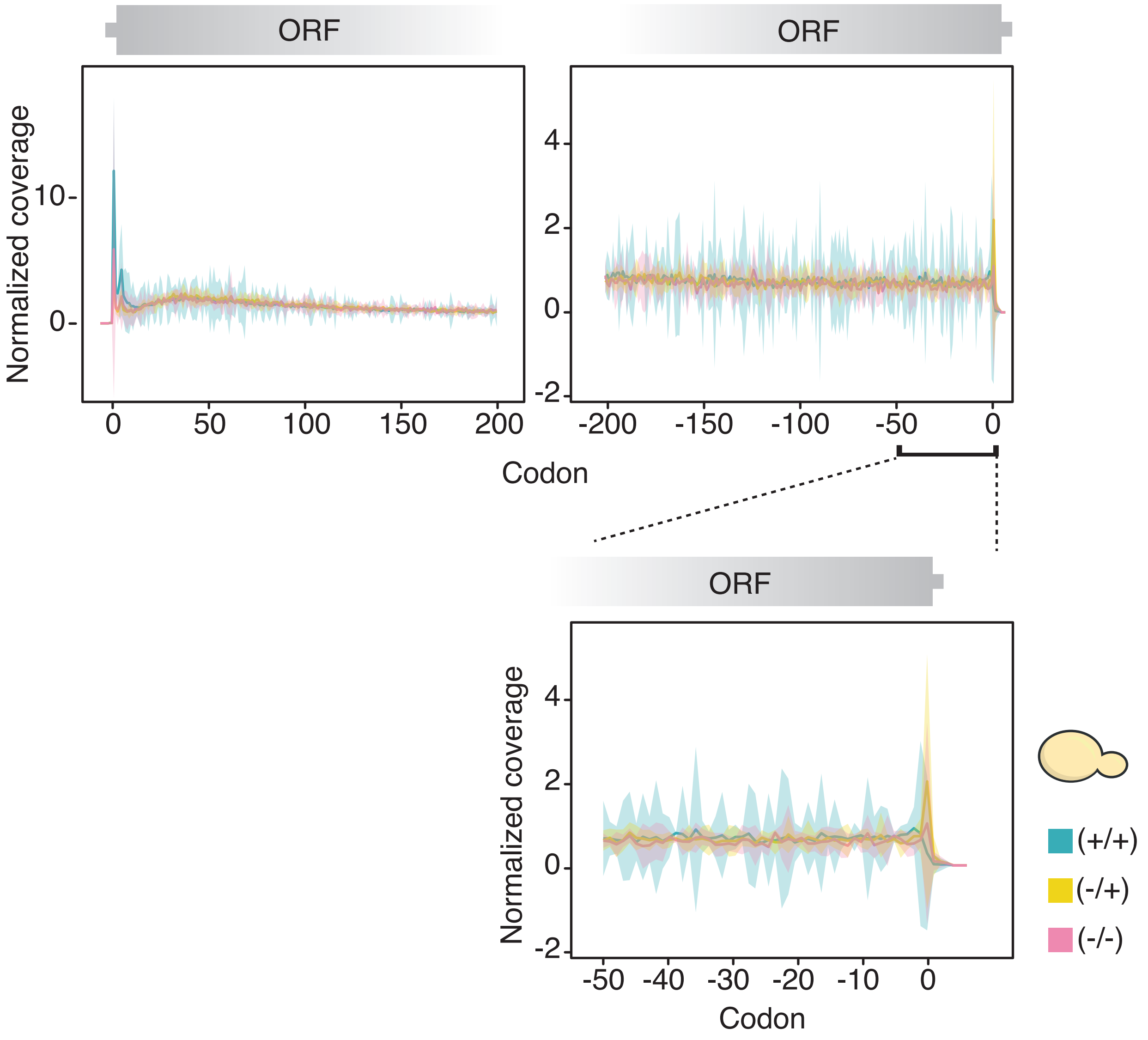

e

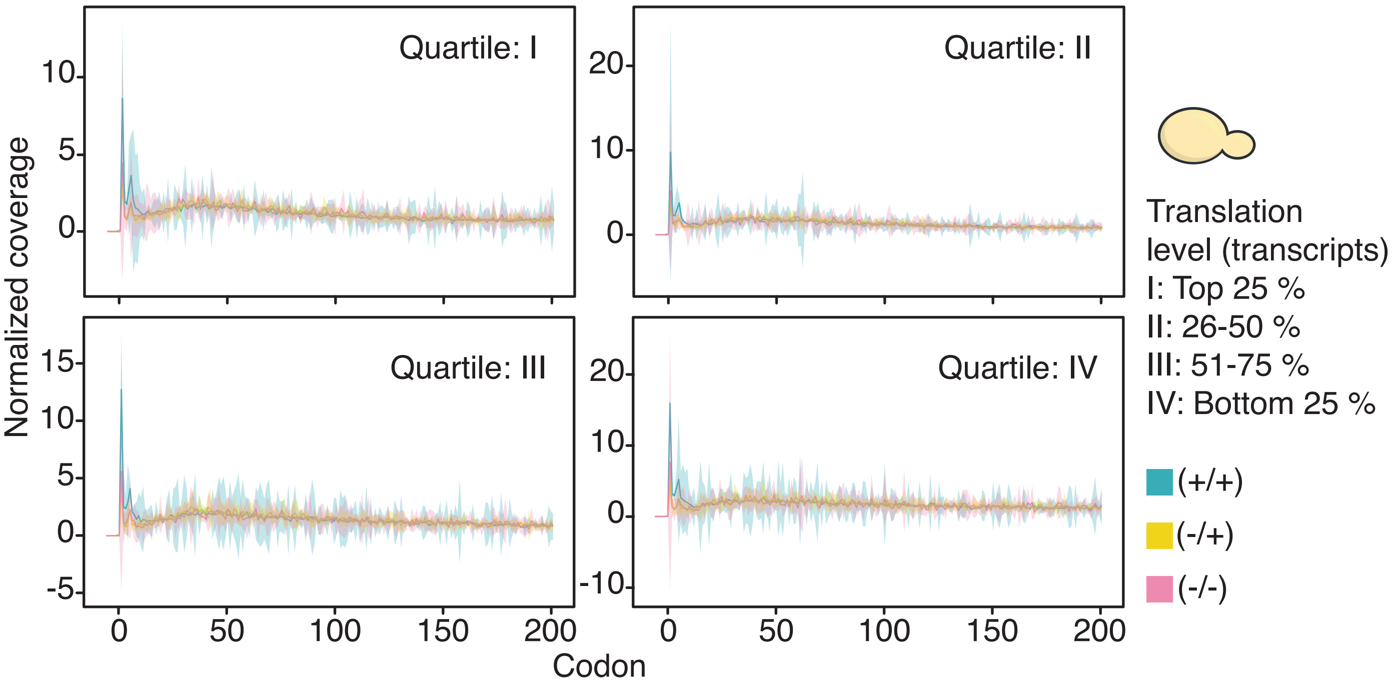

Supplementary Figure 2

f

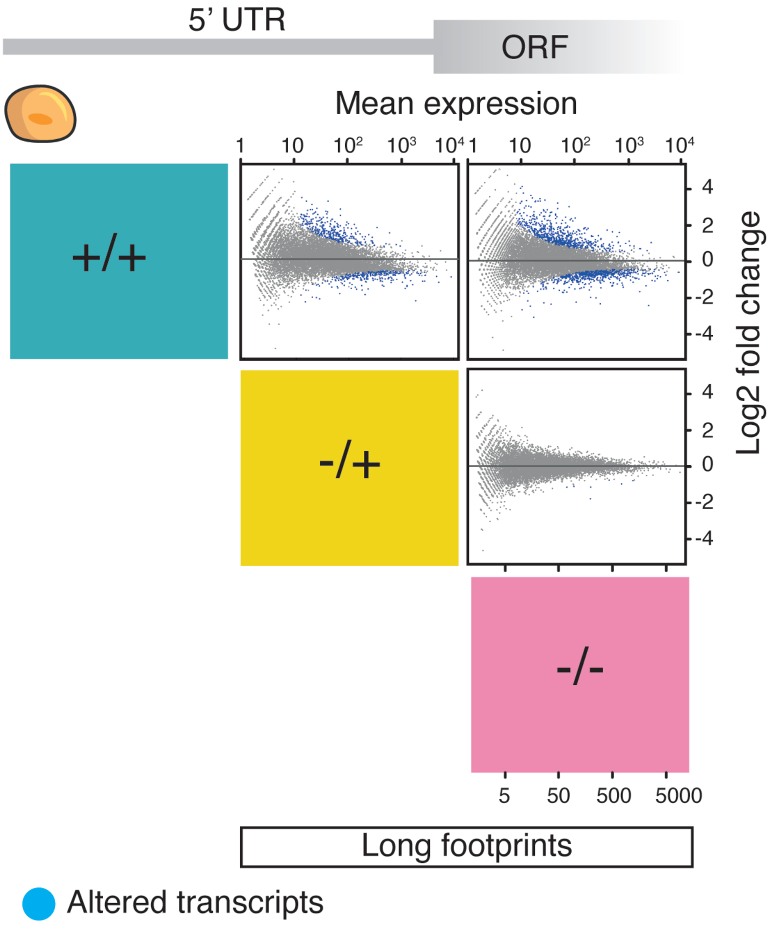

5’ UTR
ORF
Mean expression
1
10
10²
10³
10⁴
+/+
-/+
-/-
4
2
0
-2
-4
Log2 fold change
5
50
500
5000
Long footprints
Altered transcripts

Supplementary Fig. 2: Ribosome density across ORFs is independent of translation levels and gene length. a) Normalized ribosomal A-site coverage observed in short footprints (21-22 nt) for the first 200 (left) and last 200 (right) codons in HEK 293T cells in highly expressed genes (>64 reads). b) Normalized ribosomal A-site coverage for long footprints of HEK 293T cells. ORFs were divided into quartiles based on translation levels (quartile I: 25% most expressed genes, quartile II: 26%-50% most expressed genes, etc.). c) Normalized ribosomal A-site coverage for long footprints of human ORFs divided into four groups based on gene length (only genes > 200 codons). d) Normalized ribosomal A-site coverage for long footprints (27-29 nt) for the first 200 (left) and last 200 (right) codons in yeast cells in highly expressed genes (>64 reads). (Bottom) Normalized ribosomal A-site coverage of the last 50 codons in yeast cells. e) Same as b) for yeast cells. Solid line depicts the mean and shaded areas represent 95 % confidence intervals for three biological replicates (n=3). CHX-treatment conditions are indicated by color: +/+, green; -/+, yellow; -/-, pink. f) Differential ribosome occupancy of 5' UTRs in HEK 293T cells across inhibitor treatments determined with DESeq2[5]. 5' UTRs were tested for differential translation (top right; adjusted p-value ≤ 0.05). Significantly altered 5' UTRs are indicated in blue.

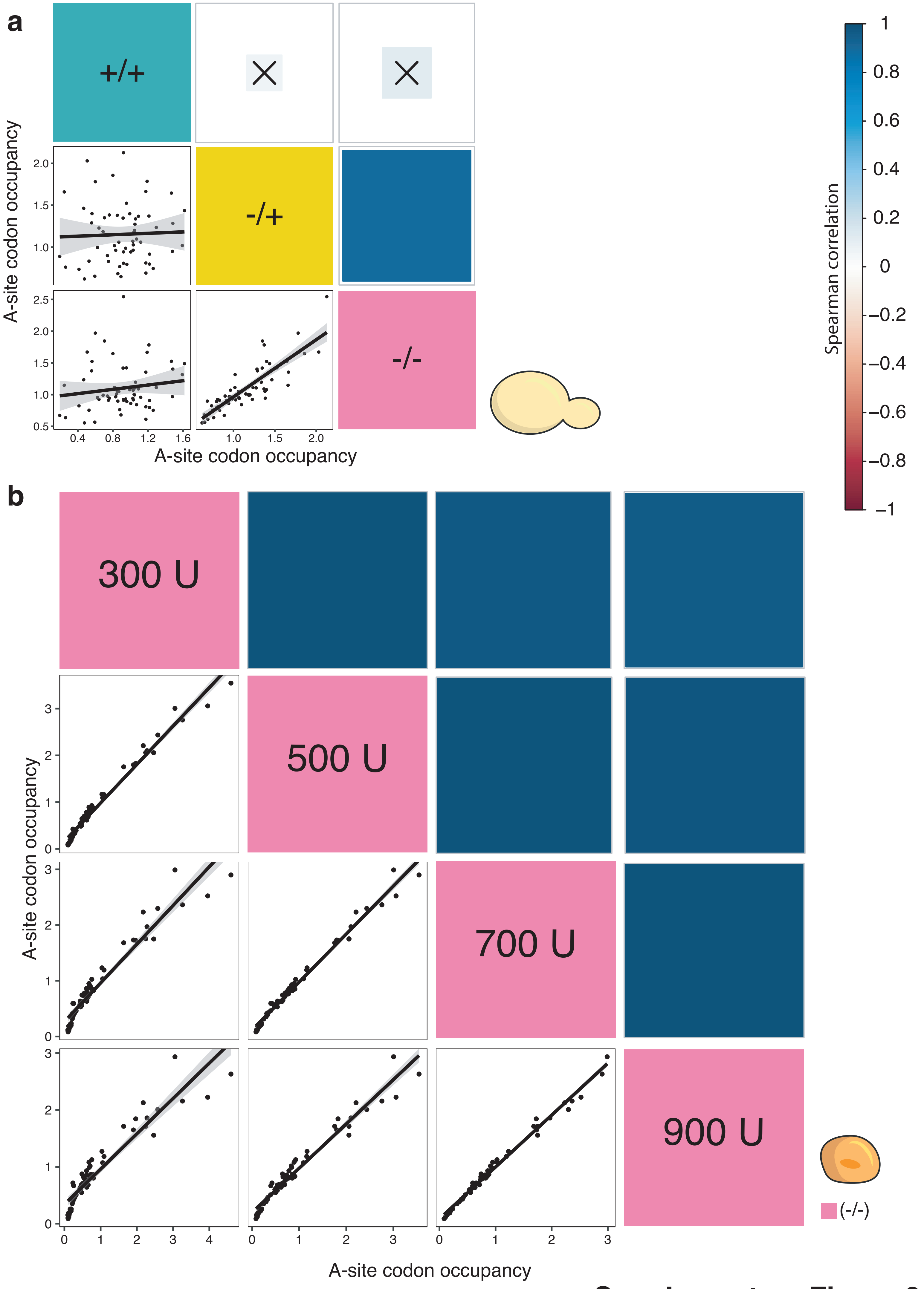

Supplementary Figure 3

c

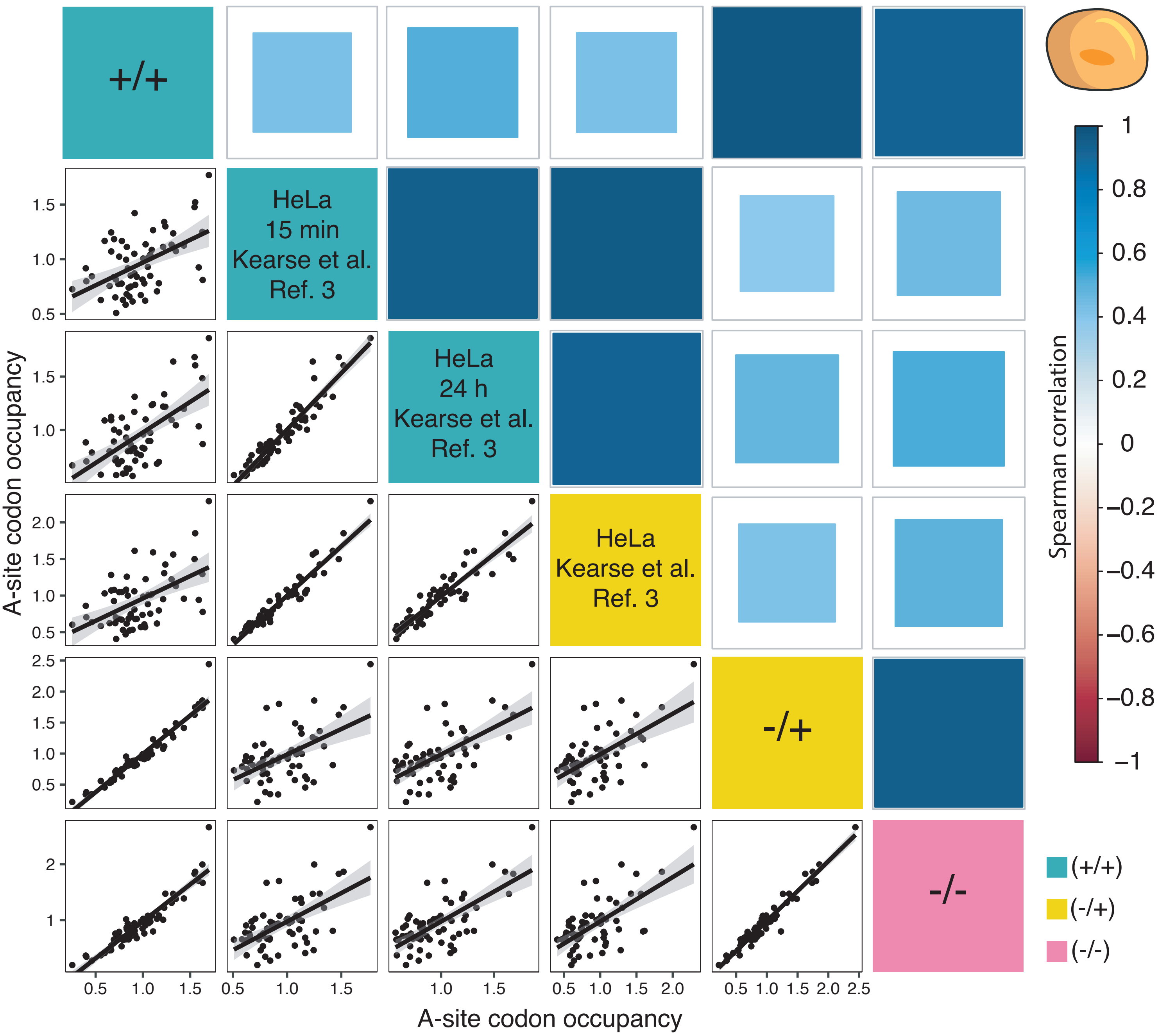

Supplementary Figure 3

d

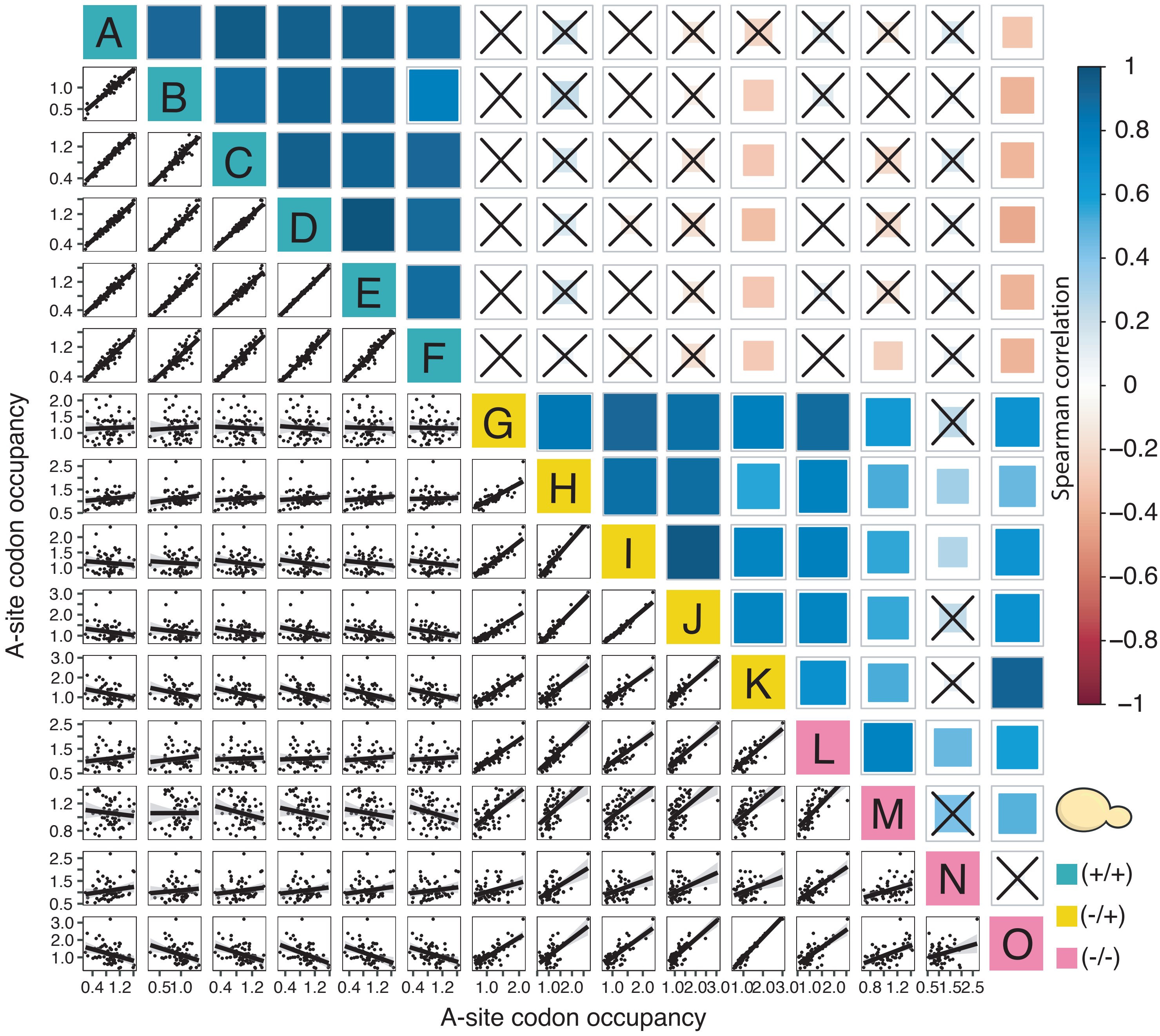

Supplementary Figure 3

e

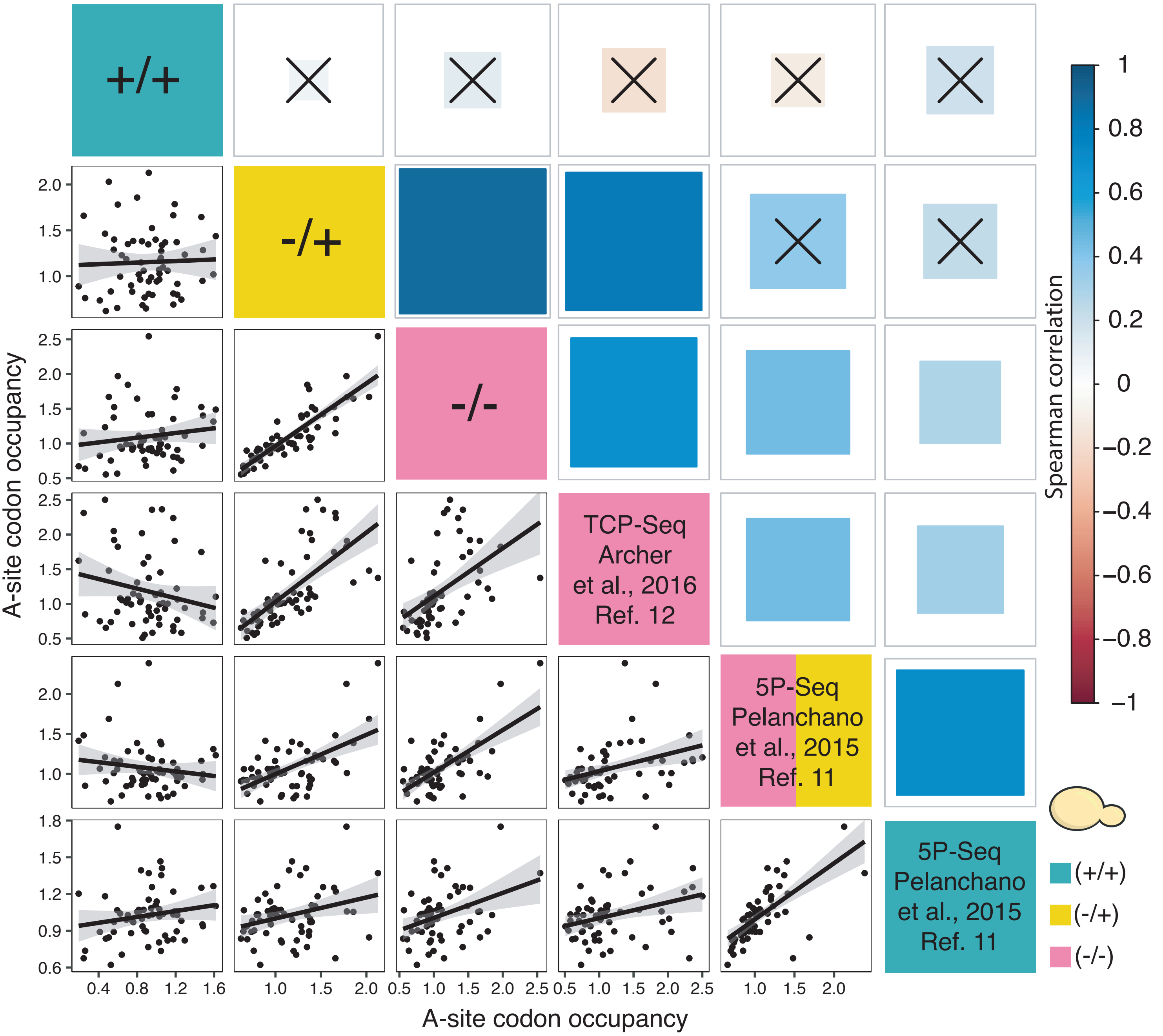

Supplementary Figure 3

f

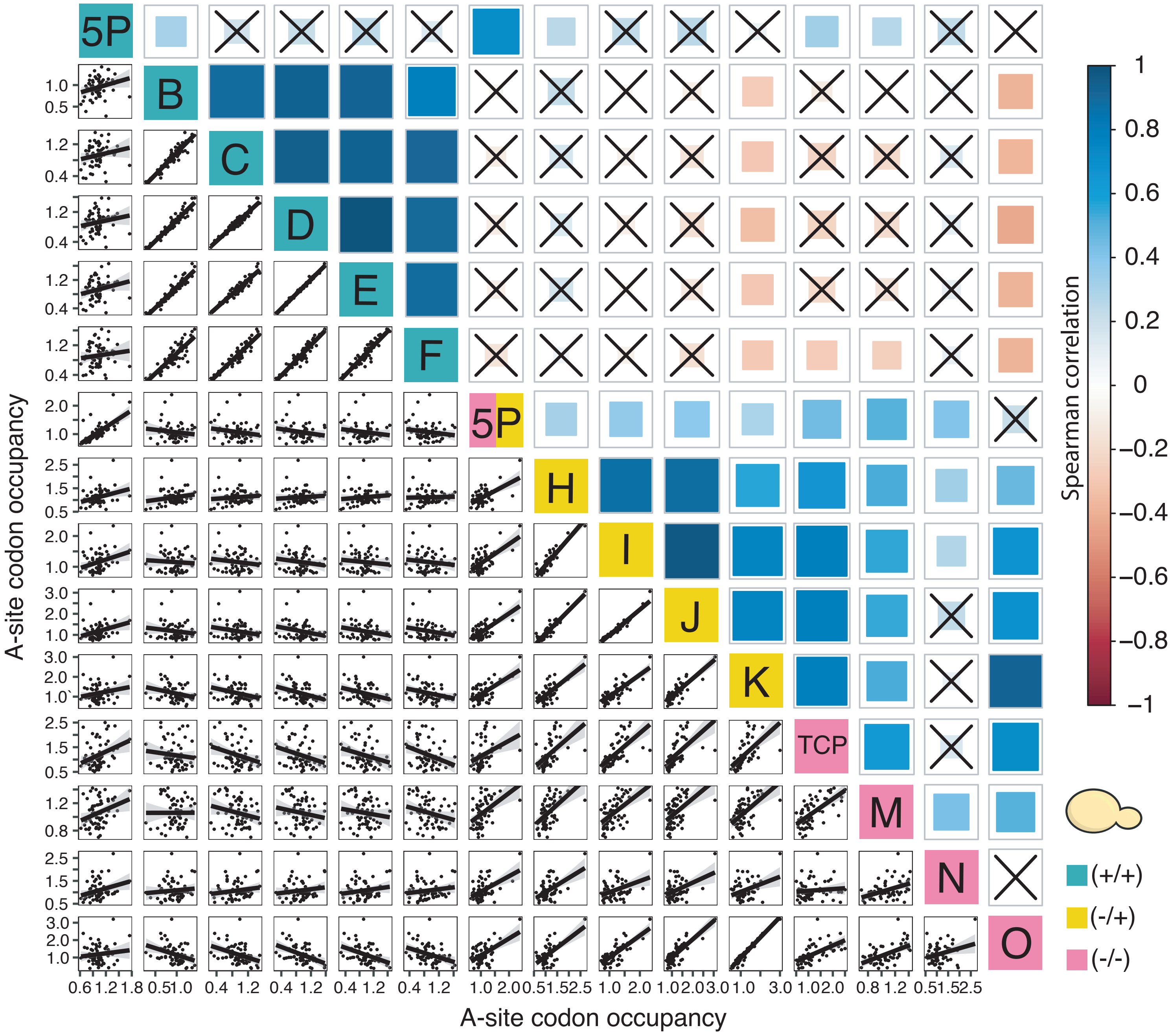

Supplementary Figure 3

Supplementary Fig. 3: Effects of different treatments on A-site codon occupancy. a) Spearman correlations of A-site ribosome occupancy in yeast cells across different CHX treatments (mean, n=3). Solid line depicts the fitted line and shaded areas represent 95 % confidence intervals for three biological replicates (n=3). Each black dot represents a codon. The size of the box indicates p-value. Correlations with a p-value > 0.05 are crossed out. b) Same as a) for -/- HEK 293T cells treated with different RNase I concentrations (n = 1). c) Same as a) for HEK 293T cells from this study and HeLa cells[3]. CHX-treatment conditions are indicated by color: +/+, green; -/+, yellow; -/-, pink. d) Same as a) across multiple yeast ribosome profiling datasets using the three regimens of CHX treatment from this study and others[1, 5, 6, 12-17]. A: This study; B:[1] ; C:[6] ; D:[7] ; E:[8]; F:[9]; G: This study; H:[6] ; I:[7] ; J:[8] ; K:[10] ; L: This study; M:[1] ; N:[9] ; O:[10]. e) Same as a) for yeast libraries generated in this study, 5PSeq[11] and TCP-seq samples[12]. f) Same as d) but including 5PSeq and TCP-seq samples. CHX-treatment conditions are indicated by color: +/+, green; -/+, yellow; -/-, pink. Note that in the 5PSeq protocol cells are harvested and RNA is immediately extracted without incubation in a lysis buffer. Therefore, 5PSeq libraries are +CHX (green) or -CHX (yellow/pink).

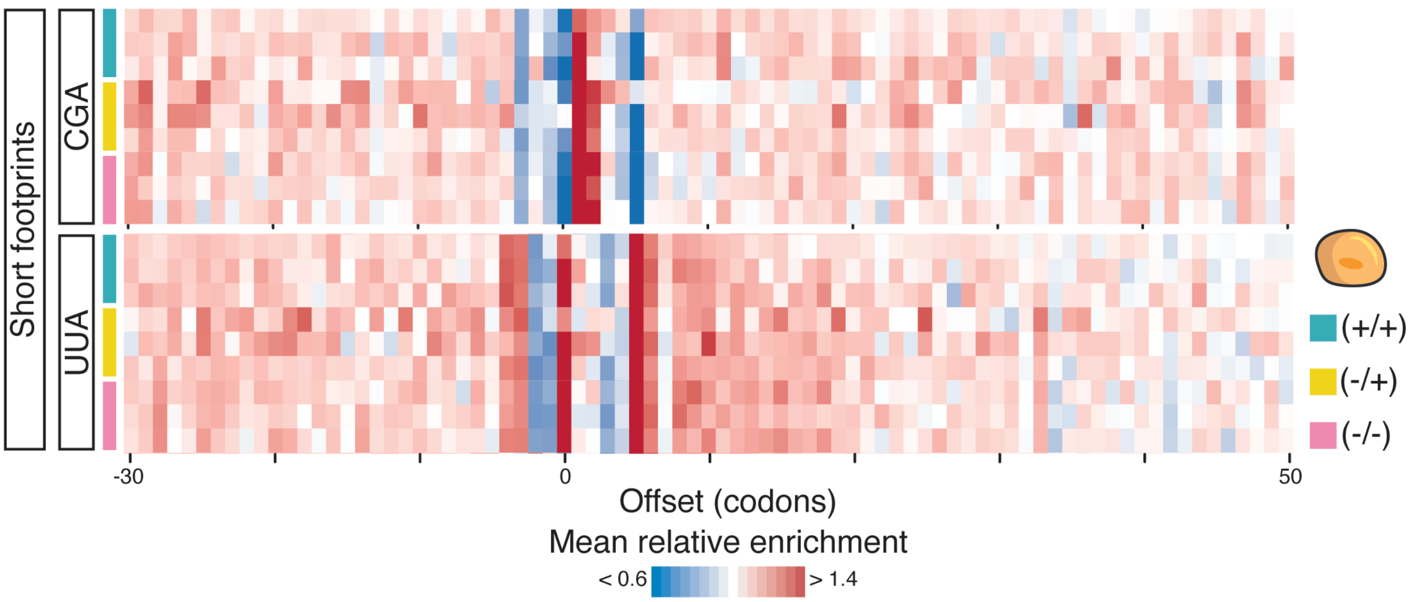

Supplementary Figure 4

Supplementary Fig. 4: Cycloheximide (CHX) pre-treatment does not alter ribosome occupancy downstream of rare codons. Transcriptome-wide ribosome enrichment profiles surrounding CGA and UUA codons according to Hussmann *et al.*[13] for short footprints (21 and 22 nt) of HEK 293T cells using different CHX treatment regimens.

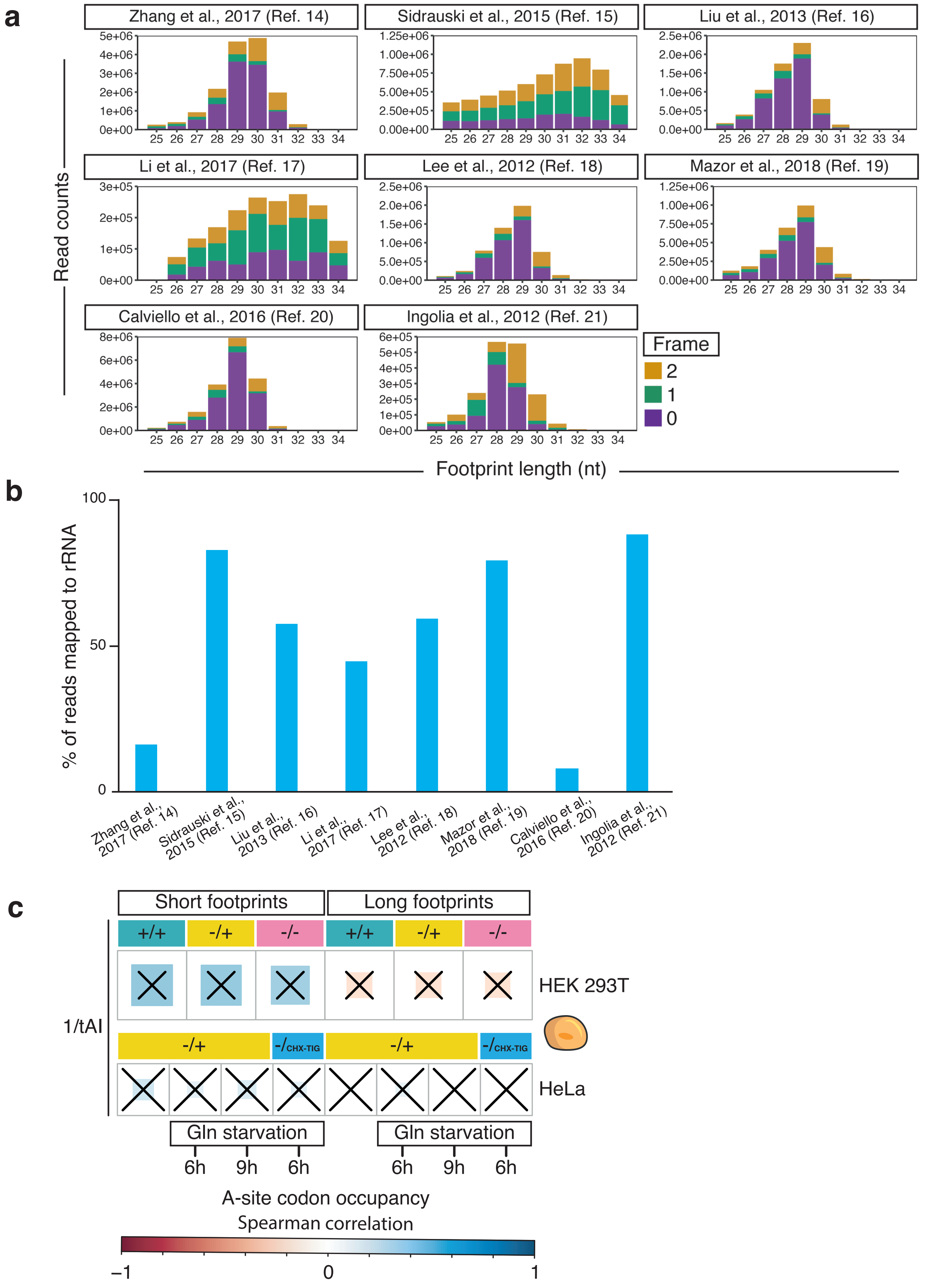

Supplementary Figure 5

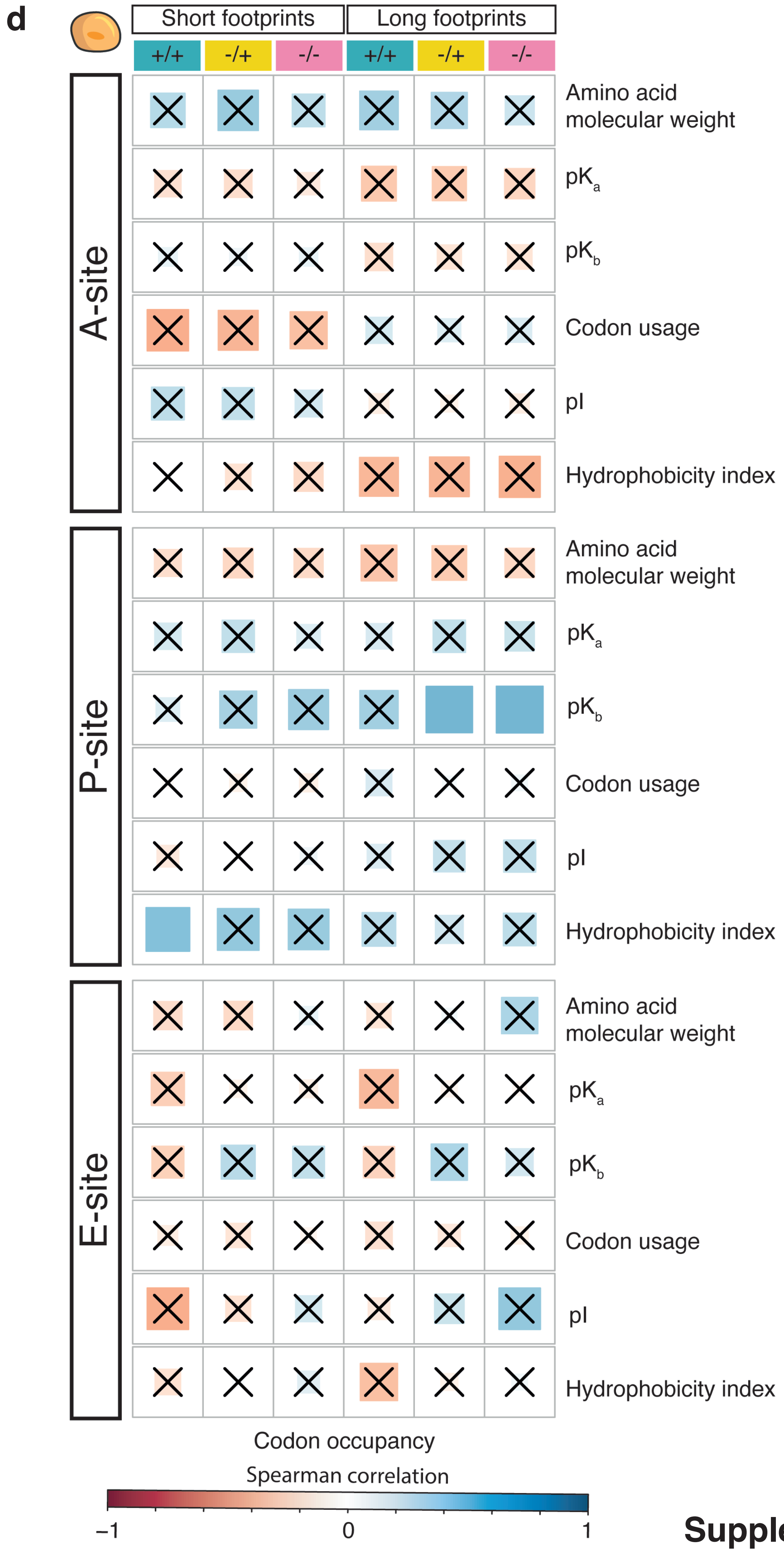

Supplementary Figure 5

Supplementary Fig. 5: Differences in footprint length, reading frame and rRNA abundance in published human cell datasets. a) Histograms showing the footprint length and the reading frame in published human libraries[14–21]. The reading frame is indicated by color: 0, purple; 1, green; 2, yellow. b) Percentage of reads mapped to rRNA in the datasets used in a). c) Spearman rank correlation of A-site codon occupancy with tRNA adaptation index for HeLa[4] and HEK 293T cells for short and long footprints. The size of the box indicates p-values. Correlations with a p-value > 0.05 are crossed out. d) Spearman rank correlation of A-, P- and E-sites codon occupancy with various amino acid properties and codon usage in HEK 293T cells for short and long footprints. The size of the box indicates p-values. Correlations with a p-value > 0.05 are crossed out.

Supplementary Table 1

	Frequency: per thousand						Copy number						
Codon	Human (1)	Mouse (2)	Zebrafish (3)	*S. cerevisiae (4)*	*C. albicans (5)*	*S. pombe (6)*	Anti-codon(s)	Human (7)	Mouse (8)	Zebrafish (9)	*S. cerevisiae (10)*	*C. albicans (11)*	*S. pombe (12)*
UUA	7.7	6.7	7.0	26.2	36.1	26.3	UAA	4	4	33	7	5	2
CUA	7.2	8.1	6.2	13.4	4.4	8.7	IAG, UAG	9, 3	5, 3	241, 193	0, 3	2, 0	5, 1
AUA	7.1	7.4	7.7	17.8	14.4	13.5	IAU, UAU	14, 5	11, 4	196, 92	13, 2	5, 1	8, 1
GUA	7.1	7.4	6.7	11.8	8.6	12.4	IAC, UAC	9, 5	7, 3	238, 113	14, 2	6, 1	9, 2
UCG	4.4	4.2	5.6	8.6	6.8	8.1	CGA, UGA	4, 4	3, 3	48, 177	1, 3	1, 3	1, 2
CCG	6.9	6.2	8.2	5.3	2.7	4.6	CGG, UGG	4, 7	3, 8	92, 159	0, 10	0, 5	1, 2
ACG	6.1	5.6	7.4	8.0	3.9	6.6	CGU, UGU	5, 6	4, 4	25, 25	1, 4	1, 3	1, 2
GCG	7.4	6.4	8.6	6.2	2.1	5.4	CGC, UGC	4, 8	9, 11	47, 173	0, 5	0, 2	1, 2
CGA	6.2	6.6	6.7	3.0	4.1	8.0	ICG, UCG	7, 6	6, 5	138, 44	6, 0	2, 0	8, 1
CGU	4.5	4.7	6.9	6.4	6.0	15.6	ICG, GCG, ACG	7, 0, 7	6, 0, 6	138, 0, 138	6, 0, 6	2, 0, 2	8, 0, 8
CGC	10.4	9.4	9.6	2.6	0.8	6.0	ICG, GCG	7, 0	6, 0	138, 0	6, 0	2, 0	8, 0
CGG	11.4	10.2	6.6	1.7	1.0	3.0	CCG, UCG	4, 6	3, 5	29, 44	1, 0	1, 0	1, 1

Source:
1 www.kazusa.or.jp/codon/cgi-bin/showcodon.cgi?species=9606
2 www.kazusa.or.jp/codon/cgi-bin/showcodon.cgi?species=10090
3 www.kazusa.or.jp/codon/cgi-bin/showcodon.cgi?species=7955
4 www.kazusa.or.jp/codon/cgi-bin/showcodon.cgi?species=4932
5 www.kazusa.or.jp/codon/cgi-bin/showcodon.cgi?species=5476
6 https://www.kazusa.or.jp/codon/cgi-bin/showcodon.cgi?species=4896
7 gtrnadb.ucsc.edu/genomes/eukaryota/Hsapi19/
8 gtrnadb.ucsc.edu/genomes/eukaryota/Mmusc10/
9 gtrnadb.ucsc.edu/GtRNAdb2/genomes/eukaryota/Dreri11/
10 gtrnadb.ucsc.edu/GtRNAdb2/genomes/eukaryota/Scere3/
11 www.candidagenome.org/
12 gtrnadb.ucsc.edu/GtRNAdb2/genomes/eukaryota/Schi_pomb_972h/

Supplementary Table 2

Study	SRR	Cell line	Cycloheximide (CHX)		rRNA	Nuclease digestion			Note	Lysis buffer
			Incubation (min)	Lysis buffer	%	Duration (min)	Temp	RNAse I		
Ingolia *et al.*, 2012 (Ref. X)	SRR493747 SRR493748	HEK293	?	+	88.22	45	Room temp	7.5 μl / 300 μl lysate		20 mM Tris·Cl (pH 7.4), 150 mM NaCl, 5 mM MgCl2, 1 mM DTT and 100 μg ml−1 cycloheximide plus 1% (vol/vol) Triton X-100 and 25 U ml 1 Turbo DNase I
Sidrauski *et al.*, 2015 (Ref. Y)	SRR1795425 SRR1795427	HEK293T	2	+	83.07	NA	NA	NA	CHX incubation in PBS not in media; The amount of RNase I and time of incubation was optimized for each sample based on the collapse of polyribosomes to the monosome peak as analyzed by analytical polyribosome gradients.	20 mM Tris pH = 7.4 (RT), 200 mM NaCl, 15 mM MgCl, 1 mM DTT, 8% glycerol, 100 μg/ml CHX, 1% Triton and protease inhibitors
Mazor *et al.*, 2018 (Ref. Y)	SRR6932676	HEK293T	3	+	79.44	NA	NA	NA	DNA libraries were constructed from the ribosome-protected mRNA fragments by poly(A) tailing and reverse transcription using barcode-containing oligonucleotides.	Polysome lysis buffer (10 mM HEPES, pH 7.4, 100 mM KCl, 5 mM MgCl2) containing cycloheximide (100 μg/mL) and Triton-X100 (1% v/v)
Li *et al.*, 2017 (Ref. Y)	SRR5882586	HEK293T	7	+	44.84	20 min	25 °C	1 μl* / 25 A260 U	*Micrococcal nuclease. RNA samples were dephosphorylated with PNK (NEB) and ligated to 3′ RP linker (5′rAPP-CTGTAGGCACCATCAAT-3SpC3) using T4 RNA ligase2, truncated KQ (NEB)	10 mM Tris-HCl, pH 7.4, 5 mM $MgCl_2$, 100 mM KCl, 1% Triton X-100, 2 mM DTT, 100 μg/mL CHX, 0.5 U/μL RNase inhibitor, 1 × complete protease inhibitor
Lee *et al.*, 2012 (Ref. Y)	SRR618770 SRR618771	HEK293	30	+	59.35	60 min	4 °C	200 U / 140 μl lysate	To remove adaptor sequences, seven nucleotides were removed from the 3′end of each 50-nt-long Illumina sequence read, and a stretch of As were removed from the 3' end, allowing one mismatch. The remaining insert sequence was separated according to the 2 nt barcode at the 5' end after the barcode was removed. Reads between 26 and 29 nt in length were mapped to the sense strand of the entire human or mouse RefSeq transcript sequence library.	Polysome lysis buffer(pH 7.4, 10 mM Hepes, 100 mM KCl, 5 mM MgCl2,100μg/mLCHX, and 2% Triton X-100)
Liu *et al.*, 2013 (Ref. Y)	SRR619083	HEK293	3	+	57.68	60 min	4 °C	200 U / 200 μl lysate		lysis buffer (10mM Tris, 50mM HEPES, 100mM KCl, 5mM MgCl2, 10% glycerol, 0.1% TritonX-100, 1mM DTT, EDTA-free protease inhibitor cocktail, pH 7.4)
Calviello *et al.*, 2015 (Ref. Y)	SRR2433794	HEK293	-	+	8.04	45 min	Room temp	3 µl RNase I / 120 µl lysate	Library generation using 3′ adaptor NN-RA3, 5′ adaptor OR5-NN, RT primer RTP and PCR primers RP1 (forward primer) and RPI6-7 (reverse primer, containing barcodes). NN-RA3, P NNTGGAATTCTCGGGTGCCAAGG-InvdT; OR5-NN, 5′-GUUCAGAGUUCUACAGUCCGACGAUCNN.	Mammalian polysome buffer (20 mM Tris-HCl, pH 7.4, 150 mM NaCl, 5 mM MgCl2, with 1 mM DTT and 100 µg/ml cycloheximide added freshly) supplemented with 1% (vol/vol) Triton X-100 and 25 U/ml Turbo DNase
Zhang *et al.*, 2017 (Ref. Y)	SRR5227448 SRR5227449	HEK293	1	+	16.17	45 min	Room temp	1500 U / 600 μl lysate	TruSeq Ribo Profile 3′ Adapter: 5′ AGATCGGAAGAGCACACGTCT	TruSeq Ribo Profile (Mammalian) Library Prep Kit (Illumina)

Supplementary Methods

Ribosome profiling protocol

The following protocol is adapted from Lecanda *et al.* 2016[2], which, itself is a modified version of Ingolia *et al.* 2012[21]. The main differences are a faster lysis method for HEK 293T cells, a modification of the RNase I digestion step and a wider ribosomal footprint-size selection. The protocol improves the proportion of in-frame mapped reads and reduces rRNA contamination in human samples.

HEK 293T cell harvesting and footprinting

HEK 293T cells were cultured in high glucose Dulbecco's modified Eagle's medium (DMEM; Gibco) supplemented with 10 % fetal bovine serum (FBS-superior; Merck) and 1x Penicillin-Streptomycin-Glutamine (Thermo Fischer Scientific) at 37 °C and 5 % CO_2. Fresh medium was provided to the cells 2 h prior to lysis. For the +/+ samples, cells were incubated with medium containing 100 µg/ml CHX for 1 min, washed with ice cold PBS and flash frozen in liquid nitrogen. The dish was swiftly transferred to ice and 400 µl lysis buffer (10 mM Tris-HCl pH 7.5, 100 mM NaCl, 5 mM $MgCl_2$, 1 % Triton X-100, 0.5 mM DTT, 0.5 % deoxycholate (w/v) and 100 µg/ml CHX) was added dropwise to the cells. The cells were harvested on ice by scraping once the lysis buffer was thawed. Cells for -/+ and -/- conditions were harvested like +/+, however, CHX pre-incubation was omitted, and it was not added to the lysis buffer for -/-. The lysate was transferred to a pre-chilled 1.5 ml microcentrifuge tube and samples were cleared by centrifugation (5 min; 4 °C; 10,000 g). A second round of clarification was performed in case that debris was observed in the lysate. The samples were aliquoted and flash-frozen in liquid nitrogen until digestion. Unless specified otherwise, 10 A_{260} units of cleared lysates were digested with 900 U RNase I (ThermoFisher) for 1 h at 22 °C with continuous agitation at 1400 rpm and the reaction was inhibited by the addition of 150 U Superase-In (ThermoFisher). To relieve ribosome aggregates 1 % (w/v) deoxycholate was added.

To isolate monosomes, samples were loaded onto linear 10–50 % (w/v) sucrose gradients (20 mM Tris-Cl (pH 7.5), 5 mM $MgCl_2$, 100 mM NH_4Cl, 1 mM DTT, 100 µg/ml Cycloheximide) prepared with Gradient Master (BioComp) and centrifuged for 3 h at 35,000 rpm, 4 °C in a Sorvall WX80 ultracentrifuge (Thermo Fischer Scientific) equipped with TH-641/SW 41 Ti rotor (Thermo Scientific/Beckman Coulter). The gradients were fractionated

using a density gradient fractionator (Isco) and a SYR-101 syringe pump (Brandel) with 60 % sucrose pushing the gradients (flow rate = 0.75 ml/min) while A_{254} was continuously monitored. 1 ml fractions corresponding to the monosome peak were collected and SDS (final concentration 1 % w/v) was immediately added to fractions, which were pooled, flash-frozen and stored at -80 °C.

Total RNA was isolated from the monosome samples using the hot-acidic-phenol method. The samples were incubated with 1 Vol acidic phenol (pH 4.3) and incubated for 5 min at 65 °C with intermittent vortexing. Subsequently, the aqueous phase was extracted with three rounds of 1 Vol acidic phenol (pH 4.3) : chloroform (5:1 ratio) extractions and one final round with 1 Vol chloroform. The total RNA was precipitated in 3 Vol absolute ethanol, 0.1 Vol NaOAc and 5 µl glycogen (Thermo Fischer Scientific) at -80 °C for at least 1 h. The same procedure was used for all RNA precipitations. The samples were centrifuged for 30 min at 4 °C, 16,000 g and the RNA pellets were resuspended in 30 µl of RNase-free water. 10 µg of total RNA from monosome fractions was mixed with RNA loading buffer (98 % (v/v) Formamide, 0.5x TBE, 0.5 % SDS) and separated using acrylamide gels (15 % polyacrylamide, 8 M Urea, 1x TBE) followed by staining with SYBR Gold (Life Technologies). Footprints were excised from acrylamide gels using RNA size markers of 18 nt (5' AUGUACACGGAGUCGACC 3') and 32 nt (5' AUGUACACGGAGUCGAGCUCAACCCGCAACGC 3'). These 18-32 nt gel slices were crushed and eluted overnight in 400 µl gel extraction buffer (300 mM NaOAc (pH 5.5), 1 mM EDTA, 10 U/ml SUPERase-In) at 4 °C with constant gentle rotation. The crushed gel pieces were removed by filtering the samples through Spin-X 0.45 µm cellulose acetate columns (Corning Costar) at 5,500 g for 1 min. The eluate was precipitated, resuspended in RNase-free water as described above and used for library generation. Note: For a standard ribosome profiling experiment only large footprints will be isolated excising 28-32 nt gel slices.

Yeast harvesting and footprinting

Overnight cultures of wild-type yeasts in the BY4741 (*S. cerevisiae*), 972(h^-) (*S. pombe*) and SN87 (*C. albicans*) backgrounds were diluted and grown to mid-exponential phase (OD_{600} ~ 0.4). For +/+ samples, CHX was added to a total concentration of 100 µg/ml and cultures were gently agitated for 1 min at 30 °C. Cells were rapidly harvested by vacuum filtration through a 0.45 µm cellulose nitrate filter (GE Healthcare) and immediately flash-frozen. Samples were mechanically lysed under cryogenic conditions in a Freezer-Mill (SPEX SamplePrep) with 2 cycles at 5 CPS interspersed by 2 min of cooling. Lysates were thawed in lysis buffer (20 mM Tris-HCl pH 7.4, 5 mM $MgCl_2$, 100 mM NaCl, 1 % Triton, 2 mM DTT)

containing 100 µg/ml CHX for +/+ and -/+ samples and clarified by two rounds of centrifugation (5 min; 4 °C; 10,000 g). Unless specified otherwise, 10 A_{260} units of cleared lysates were digested with 600 U Ambion RNase I (ThermoFisher) for 1 h at 22 °C with continuous agitation at 1400 rpm and the reaction was inhibited by the addition of 150 U SuperaseIn (ThermoFisher). Monosome isolation, footprint size selection (18-30 nt) and RNA extraction were performed analogous to human samples. Footprint size selection was performed using RNA size markers of 18 nt (5' AUGUACACGGAGUCGACC 3') and 30 nt (5' AUGUACACGGAGUCGAGCUCAACCCGCAAC 3').

Library preparation

Libraries were generated according to protocol described in detail before[21], using 3'-adapters (5' (5rApp)NNNNCTGTAGGCACCATCAAT(3ddC) 3') that were randomized at the first 4 positions of the 5' end to minimize potential ligation biases[2,22,23]. Briefly, HEK 293T samples were treated with Ribo-Zero Gold rRNA removal kit (Illumina) according to manufacturer's instruction but using only half of the recommended amounts of reagents. The depleted samples were precipitated and resuspended in RNase-free water as described above. Prior to 3' dephosphorylation, the samples were denatured for 2 min at 80 °C. Subsequently, the samples were 3' dephosphorylated by treating 20 µl sample with 5 µl T4 PNK (NEB) and 1 µl SUPERase-In at 37 °C for 60 min. The reaction was extracted with acidic phenol (pH 4.3) : chloroform (5:1) and the RNA fragments were precipitated as described above. The 5 µl RNA fragments and randomized-linker (0.5 µg/ml) were denatured for 90 s at 80 °C followed by ligation using 1 µl T4 RNA ligase 2, truncated (NEB), 2 µl T4 RNL2 buffer, 10 µl PEG8000 and 0.5 µl SUPERase-In for 4 h at 22 °C. The samples were subsequently extracted with one round of 1 Vol acidic phenol (pH 4.3): chloroform (5:1) and final round of 1 Vol chloroform. The aqueous phase was precipitated and resuspended in RNase-free water as described above.

Samples along with 1 µl of 10 µM RT primer (5' (Phos)NNNAGATCGGAAGAGCGTCGTGTAGGGAAAGAGTGTAGATCTCGGTGG TCGC(SpC18)CACTCA(SpC18)TTCAGACGTGTGCTCTTCCGATCTATTGATGGTGCC TACAG 3'; where SpC18 = Hexa-ethyleneglycol spacer), in a total volume of 12 µl, were denatured for 5 min at 75 °C. The reaction was further supplemented with 4 µl first strand buffer (5x), 1 µl DTT (0.1 M), 1 µl dNTPs (10 mM), 1 µl SUPERase-In and 1 µl SuperScriptIII (Thermo Fischer Scientific) and incubated for 40 min at 55 °C. The reaction was stopped by incubating the sample for 15 min at 70 °C. The samples were mixed with RNA loading buffer and separated by PAGE (10 % PAA, 8 M Urea, 1x TBE) along with RT primer as control. The

gels were stained with 10,000:1 diluted SYBR Gold (Thermo Fischer Scientific) and reverse transcribed fragments larger than the RT primer were excised, crushed and incubated in 360 µl RNase-free water for 15 min at 70 °C with constant shaking at 1400 rpm. The gel slices were removed by filtering the samples through Spin-X 0.45 µm cellulose acetate columns (Corning Costar) at 5500 g for 1 min. The eluate was precipitated and resuspended in RNase-free water as described above.

The rRNA contamination from the libraries was removed using custom biotinylated DNA oligonucleotides that are complementary to the reverse transcribed rRNA. The samples (8 µl) were mixed with 200 µM biotinylated oligonucleotides (1µl from the pool) in 2x SSC and denatured for 90 s at 100 °C, cooled to 37 °C at 0.05 °C/s followed by incubation at 37 °C for 15 min. The samples were mixed with 1 Vol Streptavidin beads (Dynabeads MyOne Streptavidin C1; Thermo Fisher) and incubated at 37 °C for 15 min with agitation at 1000 rpm. The supernatant was recovered using magnetic stand (DYNAL; Invitrogen) and precipitated as described above. rRNA depleted single stranded cDNA fragments were circularized in a 20 µl reaction containing 2 µl CircLigase buffer (10x), 1 µl $MnCl_2$ (50 nM), 4 µl betaine (5 M; Sigma), 1 µl ATP and 1 µl CircLigase I (Epicentre). The reaction was incubated for 3 h at 60 °C and the enzyme was inactivated for 10 min at 80 °C.

The optimum PCR cycle number required to generate the final library was experimentally determined by performing a test PCR. A master mix consisting of 3 µl circularized DNA, 0.72 µl dNTPs (10 mM), 0.18 µl PCR forward primer (100 µM), NEB Index primer (25 µM; NEBNext Multiplex Oligos for Illumina), 7.2 µl Phusion HF buffer (5x; NEB) and 0.4 µl Phusion polymerase (NEB) in a total volume of 36 µl was split into three aliquots and amplified for different number of cycles, typically 10, 12 and 14 cycles. The reaction was mixed with 2.5 µl 6x DNA loading dye and separated on a PAGE gel (8 % PAA, 1x TBE) along with a ladder, pBR322 DNA-MspI digest (NEB). The gels were stained with 10,000:1 diluted SYBR Gold (Thermo Fischer Scientific) and the optimum cycle number was determined from the reaction showing the strongest signal for a full-length library (approximately 175 nt) with the absence of background noise derived from the amplified RT primer (approximately 101 nt).

For generating the libraries meant for multiplexed sequencing, 4.2 µl of circularized cDNA from each sample were amplified as described above using index primer with unique barcode for optimum amplification cycle number. The libraries were separated on a PAGE gel and stained with SYBR Gold as described above. The bands of interest in the gel were excised, crushed and eluted with 300 µl DNA gel extraction buffer (300 nM NaCl, 10 mM Tris-Cl

(pH 7.5), 0.2 % Triton-X 100) overnight at room temperature. The gel slices were removed by filtering the samples through Spin-X 0.45 µm cellulose acetate columns (Corning Costar) at 5,500 g for 1 min. The eluate was precipitated as described above and the pellet was resuspended in EBT (0.05 % Tween, 10 mM Tris-Cl, pH 8.5). The libraries were quantified using the Qubit dsDNA HS assay (Thermo Fischer) according to manufacturer's protocol.

For sequencing, libraries were pooled based on index barcodes at a concentration of 2.2 nM (HEK 293T) or 2.0 nM (yeast) and sequenced on HiScanSQ (Illumina) generating 50 nt single end reads.

References:

1. Nedialkova, D. D. & Leidel, S. A. Optimization of Codon Translation Rates via tRNA Modifications Maintains Proteome Integrity. *Cell* **161**, 1606–1618 (2015).
2. Lecanda, A. *et al.* Dual randomization of oligonucleotides to reduce the bias in ribosome-profiling libraries. *Methods* **107**, 89–97 (2016).
3. Kearse, M. G. *et al.* Ribosome queuing enables non-AUG translation to be resistant to multiple protein synthesis inhibitors. *Genes Dev.* **33**, 871–885 (2019).
4. Wu, C. C. C., Zinshteyn, B., Wehner, K. A. & Green, R. High-Resolution Ribosome Profiling Defines Discrete Ribosome Elongation States and Translational Regulation during Cellular Stress. *Molecular Cell* **73**, 959-970.e5 (2019).
5. Love, M. I., Huber, W. & Anders, S. Moderated estimation of fold change and dispersion for RNA-seq data with DESeq2. *Genome Biol* **15**, 550 (2014).
6. Gerashchenko, M. V. & Gladyshev, V. N. Translation inhibitors cause abnormalities in ribosome profiling experiments. *Nucleic Acids Research* **42**, e134–e134 (2014).
7. Jan, C. H., Williams, C. C., & Weissman, J. S. Principles of ER cotranslational translocation revealed by proximity-specific ribosome profiling. *Science* **346**, 1257521 (2014).
8. Williams, C. C., Jan, C. H. & Weissman, J. S. Targeting and plasticity of mitochondrial proteins revealed by proximity-specific ribosome profiling. *Science* **346**, 748–751 (2014).
9. Lareau, L. F., Hite, D. H., Hogan, G. J. & Brown, P. O. Distinct stages of the translation elongation cycle revealed by sequencing ribosome-protected mRNA fragments. *eLife* **3**, e01257 (2014).
10. Guydosh, N. R. & Green, R. Dom34 Rescues Ribosomes in 3′ Untranslated Regions. *Cell* **156**, 950–962 (2014).
11. Pelechano, V., Wei, W. & Steinmetz, L. M. Widespread Co-translational RNA Decay Reveals Ribosome Dynamics. *Cell* **161**, 1400–1412 (2015).
12. Archer, S. K., Shirokikh, N. E., Beilharz, T. H., & Preiss, T. Dynamics of ribosome scanning and recycling revealed by translation complex profiling. *Nature* **535**, 570–574 (2016).

13. Hussmann, J. A., Patchett, S., Johnson, A., Sawyer, S. & Press, W. H. Understanding Biases in Ribosome Profiling Experiments Reveals Signatures of Translation Dynamics in Yeast. *PLoS Genet* **11**, e1005732 (2015).

14. Zhang, P. *et al.* Genome-wide identification and differential analysis of translational initiation. *Nat Commun* **8**, 1749 (2017).

15. Sidrauski, C., McGeachy, A. M., Ingolia, N. T. & Walter, P. The small molecule ISRIB reverses the effects of eIF2α phosphorylation on translation and stress granule assembly. *eLife* **4**, e05033 (2015).

16. Liu, B., Han, Y. & Qian, S.-B. Cotranslational Response to Proteotoxic Stress by Elongation Pausing of Ribosomes. *Molecular Cell* **49**, 453–463 (2013).

17. Li, X. *et al.* Base-Resolution Mapping Reveals Distinct m1A Methylome in Nuclear- and Mitochondrial-Encoded Transcripts. *Molecular Cell* **68**, 993-1005.e9 (2017).

18. Lee, S. *et al.* Global mapping of translation initiation sites in mammalian cells at single-nucleotide resolution. *Proceedings of the National Academy of Sciences* **109**, E2424–E2432 (2012).

19. Mazor, K. M. *et al.* Effects of single amino acid deficiency on mRNA translation are markedly different for methionine versus leucine. *Sci Rep* **8**, 8076 (2018).

20. Calviello, L. *et al.* Detecting actively translated open reading frames in ribosome profiling data. *Nat Methods* **13**, 165–170 (2016).

21. Ingolia, N. T., Brar, G. A., Rouskin, S., McGeachy, A. M. & Weissman, J. S. The ribosome profiling strategy for monitoring translation in vivo by deep sequencing of ribosome-protected mRNA fragments. *Nat Protoc* **7**, 1534–1550 (2012).

22. Romaniuk, E., McLaughlin, L. W., Neilson, T. & Romaniuk, P. J. The Effect of Acceptor Oligoribonucleotide Sequence on the T4 RNA Ligase Reaction. *European Journal of Biochemistry* **125**, 639–643 (1982).

23. Hafner, M. *et al.* RNA-ligase-dependent biases in miRNA representation in deep-sequenced small RNA cDNA libraries. *RNA* **17**, 1697–1712 (2011).
