# Supplementary material for: Humans and other commonly used model organisms are resistant to cycloheximide-mediated biases in ribosome profiling experiments
Source: Nat Commun. 2021 Aug 24;12:5094. doi: 10.1038/s41467-021-25411-y (PMC8384890; doi:10.1038/s41467-021-25411-y)
Supplement: Supplementary file 3 — Description of Additional Supplementary Files [file 41467_2021_25411_MOESM3_ESM.pdf]

Description of Additional Supplementary Files

File Name: Supplementary Data 1
Description: Contains the genes with differential abundance between conditions analyzed in this study
