# Supplementary material for: Humans and other commonly used model organisms are resistant to cycloheximide-mediated biases in ribosome profiling experiments
Source: Nat Commun. 2021 Aug 24;12:5094. doi: 10.1038/s41467-021-25411-y (PMC8384890; doi:10.1038/s41467-021-25411-y)
Supplement: Supplementary file 5 — Reporting Summary [file 41467_2021_25411_MOESM5_ESM.pdf]

nature research

Corresponding author(s): Sebastian Leidel

Last updated by author(s): Jul 28, 2021

Reporting Summary

Nature Research wishes to improve the reproducibility of the work that we publish. This form provides structure for consistency and transparency in reporting. For further information on Nature Research policies, see our Editorial Policies and the Editorial Policy Checklist.

Statistics

For all statistical analyses, confirm that the following items are present in the figure legend, table legend, main text, or Methods section.

n/a	Confirmed	
[ ]	[x]	The exact sample size (n) for each experimental group/condition, given as a discrete number and unit of measurement
[ ]	[x]	A statement on whether measurements were taken from distinct samples or whether the same sample was measured repeatedly
[ ]	[x]	The statistical test(s) used AND whether they are one- or two-sided<br>*Only common tests should be described solely by name; describe more complex techniques in the Methods section.*
[ ]	[x]	A description of all covariates tested
[ ]	[x]	A description of any assumptions or corrections, such as tests of normality and adjustment for multiple comparisons
[ ]	[x]	A full description of the statistical parameters including central tendency (e.g. means) or other basic estimates (e.g. regression coefficient) AND variation (e.g. standard deviation) or associated estimates of uncertainty (e.g. confidence intervals)
[ ]	[x]	For null hypothesis testing, the test statistic (e.g. F, t, r) with confidence intervals, effect sizes, degrees of freedom and P value noted<br>*Give P values as exact values whenever suitable.*
[x]	[ ]	For Bayesian analysis, information on the choice of priors and Markov chain Monte Carlo settings
[x]	[ ]	For hierarchical and complex designs, identification of the appropriate level for tests and full reporting of outcomes
[ ]	[x]	Estimates of effect sizes (e.g. Cohen's d, Pearson's r), indicating how they were calculated

Our web collection on statistics for biologists contains articles on many of the points above.

Software and code

Policy information about availability of computer code

Data collection	Software that was used in this study include: FASTX-Toolkit (version 0.0.13), Bowtie (version 1.0.0 and 1.2.1.1), R (version 3.4.1), DESeq2 (version 1.27), corrplot (version 0.84). The description of the algorithms applied are found in the methods section. Source codes for codon occupancy calculations are available on github (https://github.com/LeidelLab/Codon_occupancy_cal).
Data analysis	No commercial software was used for data analysis

For manuscripts utilizing custom algorithms or software that are central to the research but not yet described in published literature, software must be made available to editors and reviewers. We strongly encourage code deposition in a community repository (e.g. GitHub). See the Nature Research guidelines for submitting code & software for further information.

Data

Policy information about availability of data

All manuscripts must include a data availability statement. This statement should provide the following information, where applicable:

- Accession codes, unique identifiers, or web links for publicly available datasets
- A list of figures that have associated raw data
- A description of any restrictions on data availability

All sequencing data from the ribosome profiling experiments are available at the Gene Expression Omnibus (Accession number: GSE136940). Human (hg38) , mouse (mm10) and zebrafish (danRer10) transcript annotations are available from the UCSC table browser. Annotations for C. albicans were retrieved from http://www.candidagenome.org, for S. cerevisiae from https://www.yeastgenome.org and annotations for S. pombe from EnsemblFungi.

Field-specific reporting

Please select the one below that is the best fit for your research. If you are not sure, read the appropriate sections before making your selection.

- [x] Life sciences
- [ ] Behavioural & social sciences
- [ ] Ecological, evolutionary & environmental sciences

For a reference copy of the document with all sections, see nature.com/documents/nr-reporting-summary-flat.pdf

Life sciences study design

All studies must disclose on these points even when the disclosure is negative.

Sample size	No statistical methods was used to determine the sample size. Generally, 2-3 replicates are considered sufficient to analyze ribosome profiling experiments in the field.
Data exclusions	For the S. cerevisiae codon correlation plot, one (+/+) library was excluded (thus, n = 2 for the +/+ condition but n = 3 for -/+ and -/-). The decision was based on frame periodicity and overall quality of the library.
Replication	We confirmed our findings in 2-3 independent biological replicates and used published data for further verification.
Randomization	Randomization of subgroups is not relevant while performing ribosome profiling experiments on different inhibitor treatments. The data analysis was automated. The manuscript does not deal with human or animal subjects.
Blinding	Blinding is not relevant to the study. Furthermore, in yeast, samples of the different treatments can be identified by their characteristics such that blinding is impossible. In humans the differences were minor, such that blinding would not have affected the outcome.

Reporting for specific materials, systems and methods

We require information from authors about some types of materials, experimental systems and methods used in many studies. Here, indicate whether each material, system or method listed is relevant to your study. If you are not sure if a list item applies to your research, read the appropriate section before selecting a response.

Materials & experimental systems

n/a	Involved in the study	
x		Antibodies
	x	Eukaryotic cell lines
x		Palaeontology and archaeology
x		Animals and other organisms
x		Human research participants
x		Clinical data
x		Dual use research of concern

Methods

n/a	Involved in the study	
x		ChIP-seq
x		Flow cytometry
x		MRI-based neuroimaging

Eukaryotic cell lines

Policy information about cell lines

Cell line source(s)	HEK 293T (in this study) - ATTC resource center<br>HEK 293 (published datasets)<br>HeLa (published datasets)
Authentication	The cells were originally purchased from the ATTC resource center. The cell line was not authenticated.
Mycoplasma contamination	HEK 293T cells used in this study were tested negative. Published datasets were not analyzed for Mycoplasma contamination by us.
Commonly misidentified lines (See ICLAC register)	No commonly misidentified cell lines were used in the study.
